# Inhibition of ERK signaling for treatment of ERRα positive TNBC

**DOI:** 10.1371/journal.pone.0283047

**Published:** 2023-05-10

**Authors:** David Musheyev, Esther Miller, Natania Birnbaum, Elisheva Miller, Shoshana Erblich, Alyssa Schuck, Anya Alayev

**Affiliations:** 1 Department of Internal Medicine, Stony Brook Southampton Hospital, Southampton, New York, United States of America; 2 Department of Biology, Stern College for Women, Yeshiva University, New York, New York, United States of America; 3 Department of Mechanical Engineering, Rutgers University, New Brunswick, New Jersey, United States of America; Institute of Biomedical Sciences, TAIWAN

## Abstract

Breast cancer is the second leading cause of cancer-related deaths in women and triple-negative breast cancer (TNBC), in particular, is an aggressive and highly metastatic type of breast cancer that does not respond to established targeted therapies and is associated with poor prognosis and worse survival. Previous studies identified a subgroup of triple-negative breast cancer patients with high expression of estrogen related receptor alpha (ERRα) that has better prognosis when treated with tamoxifen. We therefore set out to identify common targets of tamoxifen and ERRα in the context of TNBC using phosphoproteomic analysis. In this study, we discovered that phosphorylation of mitogen-activated protein kinase 1 (MAPK1) is regulated by tamoxifen as well as ERRα. Additionally, we showed that inhibition of MAPK signaling together with the use of a selective ERRα inverse agonist, XCT-790, leads to a significant upregulation of apoptosis and paves way for the therapeutic use of MAPK inhibitors for treatment of ERRα expressing TNBC.

## Introduction

Breast cancer is one of the most common cancers in women and is the second leading cause of cancer-related deaths in women [1, 2]. The rate at which breast cancer is diagnosed is astonishing, with about a quarter of a million women diagnosed with invasive breast cancer in the United States annually and 1 in 8 women diagnosed within their lifetime [3]. Risk factors such as age, body-mass-index, and ethnicity are confounding factors for developing breast cancer [3–6]. Certain ethnic groups are disproportionately affected by breast cancer with a higher mortality despite a lower incidence, indicating that there remains a lot about this disease that we still do not know [3–5].

Further subclassification of breast cancer based on molecular status of the tumor reveals a correlation between prognosis and receptor expression. Tumors are classified based on expression of the following known molecular markers: estrogen receptor alpha (ERα), progesterone receptor (PR), and human epidermal growth factor receptor 2 (HER2) [5, 7]. ERα expressing tumors account for 70% of breast cancers [8] and carry a better prognosis than triple negative breast cancer (TNBC), classified as tumors that do not express ERα, PR and no amplification of HER2, which account for 15–20% of breast cancers [5, 9–12]. Unlike ERα expressing tumors, TNBCs tend to affect younger, premenopausal women, have a higher mortality rates of 40% within the first 5 years after diagnosis, and disproportionately affect African Americans [3, 5]. A combination of the poor prognosis in TNBC and predominance in African Americans might be driving the racial disparity of breast cancer survival. Therefore, the difference in prognosis and receptor expression between ERα expressing tumors and TNBCs requires diverging therapeutic approaches in the treatment of the two cancers.

Approaches to treating ERα expressing tumors focus on inhibiting ERα function with endocrine therapies by either antagonizing the binding of estrogen to ERα (selective estrogen receptor modulators), promoting ERα degradation (selective estrogen receptor degraders), or by blocking estrogen synthesis (aromatase inhibitors) [13, 14]. An example of one of the earlier used selective estrogen receptor modulators (SERM) is tamoxifen. Approved by the FDA in 1977, it was found to be most beneficial in tumors with high expression of ERα and continues to be used therapeutically today [14, 15]. Patients respond very well to tamoxifen treatment with 40–50% reduction in both distant and local recurrence with 5 years of treatment [8]. However, new or acquired resistance develops in approximately 30% of cases and tumors can spontaneously convert to hormone-independent proliferation or can lose ERα expression altogether, creating a greater need for understanding molecular mechanisms of hormone receptor negative breast cancer [15–18]. Therefore, further research has focused on identifying alternative molecular pathways responsible for tumor progression as druggable targets [9, 10, 12, 19], and such research overlaps with the search for targeted treatments for TNBCs.

Since TNBC tumors do not express any known markers, treatment options for TNBC patients are extremely limited and currently no targeted therapies exist for such patients [12]. This leaves chemotherapy as the only treatment option for TNBC [11, 12, 20–22] with a three-year overall survival of 74% compared to 89% in non-TNBC tumors, indicating that chemotherapy is not very effective [20]. Besides for leading to a significantly lower survival in ERα negative tumors as compared to ERα expressing tumors, this treatment option burdens patients with the adverse effects of chemo-toxicity such as alopecia, myelosuppression, gastrointestinal disturbances, nephrotoxicity, neurotoxicity, cardiotoxicity, and infertility, calling into question any benefit from chemotherapy at the cost of a reduced quality of life [23]. Current scientific work aims to offer insights into molecular drivers that may be leveraged in the treatment of TNBC by further classifying tumor microenvironment, identifying tumor cellular signatures and mRNA expression profiles [9, 10, 12, 19]. One such molecular driver that is currently explored is estrogen related receptor α (ERRα) [24, 25].

ERRα is an orphan nuclear receptor that is part of the superfamily of transcription factors, which include ERRα, ERRβ, ERRγ [26]. ERRα is structurally most similar to ERα and there is an overlap in ERα and ERRα binding to response elements in promoters of genes whose expression they regulate [27]. Despite their homology, ligands, such as estrogen, that bind to ERα do not bind to ERRα, therefore signaling between the two molecules is quite divergent [27]. Though its function in metabolic processes in the muscle heart and liver has been described, its role in tumorigenesis is not fully understood [28–32]. Metabolic functions that are regulated or that are thought to influence ERRα include glycolysis, cholesterol metabolism, fatty acid oxidation, and oxidative metabolism [28, 31]. In breast cancer, ERRα expression is mutually exclusive with ERα expression and is corelated with a more aggressive and metastatic disease [27]. Previous studies have shown that although ERRα was a negative predictive marker for progression free survival and disease recurrence, in TNBC-basal-like tumors, when treated with tamoxifen, ERRα expression was associated with a slightly prolonged distal metastasis-free survival, while those treated with chemotherapy alone, had significantly shorter interval to metastasis. Additionally, patients with elevated nuclear expression of ERRα who were treated with tamoxifen had a better prognosis than patients with elevated nuclear ERRα who were not treated with tamoxifen, indicating that there is a therapeutic benefit to treating ERRα-expressing TNBC patients with tamoxifen and that the action of tamoxifen in TNBCs is ERRα dependent [25]. These findings indicated that a subgroup of ERα-negative patients respond to tamoxifen and this effect is dependent on ERRα expression. This evidence prompted our current study, whose aim was to investigate the relationship between ERRα and tamoxifen in the context of TNBC. To investigate this relationship, phosphoproteomic analysis was performed in TNBC cells to identify common pathways that are regulated by both tamoxifen as well as XCT-790, an inverse agonist of ERRα [33] and to identify therapeutic targets. Our findings identify MAPK1 (also known as ERK2) as a common target for tamoxifen and ERRα and show that treatment of TNBC cells with a MEK1/2 inhibitor together with XCT-790 leads to activation of apoptosis and inhibition of cell migration and invasion, suggesting that ERK is a potential novel therapeutic target that should be considered for TNBC treatment.

## Materials and methods

### Cell culture and treatments

MDA-MB-231, MDA-MB-436, MDA-MB-157, Hs 578T, BT-549 sh-control and sh-ERRα cells were grown in Dulbecco’s modified Eagle’s medium (Gibco) with 10% fetal bovine serum (R&D, a Bio-Techne Brand) and 1% penicillin–streptomycin (Gibco). Cells were cultured in a 37°C incubator with a humidified 5% CO2 atmosphere. MDA-MB-231, MDA-MB-436, MDA-MB-157, Hs 578T, BT-549 cells were purchased from ATCC. sh-ERRα and sh-control cells were a kind gift from Dr. Marina Holz.

Cells were treated with either 100 nM tamoxifen in ethanol (Millipore), 10 μM U0126 in DMSO (Tocris, a Bio-Techne Brand), or 10 μM XCT-790 in DMSO (Tocris, a Bio-Techne Brand), alone or in combination.

### Immunoblotting

Following treatment, cells were lysed in ice cold lysis buffer (RIPA buffer with Triton^®^X-100, Halt^™^ Protease & Phosphatase Single-Use Inhibitor Cocktail (100X), ThermoScientific). Insoluble materials were centrifuged out at 14,000 rpm and 4°C for 10 min. Using the Bradford assay (Coomassie Protein Assay Reagent, ThermoScientific) and the Eppendorf BioPhotometer, cell protein concentrations were measured and normalized. 4X LDS Sample Buffer (Invitrogen B0008) and Bolt^™^ 10x Sample Reducing Agent (Invitrogen) were added to the samples followed by denaturation for 10 minutes at 70°C. Samples were resolved through electrophoresis using NuPage^™^ 4–12% Bis-Tris Gels (Invitrogen) and then transferred onto a nitrocellulose membrane (ThermoScientific) for staining. Immunoblots were detected using the following primary antibodies: ERRα #13826, Phospho-p44/42 MAPK (ERK1) (Tyr204)/(ERK2) (Tyr187) #5726, Phospho-p90RSK1 (Ser380) #12032, RSK1 #8408, p44/42 MAPK (ERK1/2) #4695, Phospho-MAPK Substrates Motif [PXpTP] MultiMab^™^ #14378, Phospho-mTOR (Ser2448) #5536, PARP #9532, and β-Actin #4970. All primary antibodies were ordered from Cell Signaling Technology. After staining with primary antibodies, nitrocellulose membranes were treated with IRDye conjugated secondary antibodies (IRDye^®^ 680RD Donkey anti-Mouse IgG Secondary Antibody, IRDye^®^ 800CW Goat anti-Rabbit IgG Secondary Antibody, Li-COR), and then imaged using the Odyssey-Clx Li-COR infrared detection instrument. Quantification of immunoblots was performed using Image Studio 5.2 (Li-COR).

### Immunoprecipitation assay

Cells were lysed in ice cold IP lysis buffer (Pierce^™^ IP Lysis Buffer, Halt^™^ Protease & Phosphatase Single-Use Inhibitor Cocktail (100X), Thermo Scientific). Lysates were subjected to immunoprecipitation with either Sepharose^®^Bead Conjugated p44/42 MAPK (Erk1/2) antibody #5736 (Cell Signaling Technology) or Protein A Agarose beads, 50% slurry (EMD Millipore) overnight at 4°C. Beads were pelleted from solution, washed twice with IP buffer and once with PBS and boiled using Invitrogen’s LDS Sample Buffer and Reducing Agent according to manufacturer’s instructions.

### Phosphoproteomics sample processing and data analysis

Cells were lysed in RIPA lysis buffer, the supernatant was collected following centrifugation at 21000xg for 10 min, and an acetone precipitation was performed overnight at -20°C. The samples were re-suspended in 7 M urea, reduced with 5 mM DTT (dithiothreitol) and alkylated with 15 mM CAA (chloroacetamide). A standard tryptic digest was performed overnight at 37°C. Solid Phase Extraction (SPE) was then performed using C18 Prep Sep^™^ cartridges (Waters, WAT054960), followed by reconstitution in 0.5% TFA (trifluoroacetic acid). The SPE cartridge was washed with conditioning solution (90% methanol with 0.1% TFA), and equilibrated with 0.1% TFA. The sample was passed (1 drop/sec) through the equilibrated cartridge, then desalted. The sample was then eluted (1 drop/sec) with an elution solution (50% ACN (acetonitrile) with 0.1% TFA. The sample was then TMT labeled according to kit specifications (ThermoFisher Scientific, 90110), with the exception that labeling was performed for 6hrs instead of 1 hr. Following labeling, another SPE was performed, as stated above. Phosphopeptide enrichment using Titansphere Phos-TiO Kit (GL Sciences, 5010–21312) was then performed. Briefly, samples were reconstituted in 100 μL of Buffer B (75% ACN, 1% TFA, 20% lactic acid–solution B in the kit). The tip was conditioned by centrifugation with 100 μL of Buffer A (80% ACN, 1% TFA), followed by conditioning with Buffer B (3000xg, 2min). The samples were then loaded onto the tip and centrifuged twice (1000xg, 5min). The tip was then washed with 50 μL of Buffer B, followed by 2 washes with 50 μL of Buffer A (1000xg, 2min). The samples were eluted with 100 μL of elution 1 (20% ACN, 5% NH4OH) then 100 μL of elution 2 (20% ACN, 10% NH4OH) (1000xg, 5min). A final clean-up step was performed using C18 Spin Columns (Pierce, 89870).

### Mass spectrometry, data filtering, and bioinformatics

Mass spectrometry analysis was carried out as follows: to separate peptides, reverse-phase nano-HPLC was performed by a nanoACQUITY UPLC system (Waters Corporation). Peptides were trapped on a 2 cm column (Pepmap 100, 3 μM particle size, 100 Å pore size), and separated on a 25cm EASYspray analytical column (75 μM ID, 2.0 μm C18 particle size, 100 Å pore size) at 45°C. The mobile phases were 0.1% formic acid in water (Buffer A) and 0.1% formic acid in acetonitrile (Buffer B). A 180-minute gradient of 2–30% buffer B was used with a flow rate of 300 nl/min. Mass spectral analysis was performed by an Orbitrap Fusion Lumos mass spectrometer (ThermoFisher Scientific). The ion source was operated at 2.4kV and the ion transfer tube was set to 275oC. Full MS scans (350–2000 m/z) were analyzed in the Orbitrap at a resolution of 120,000 and 4e5 AGC target. The MS2 spectra were collected using a 0.7 m/z isolation width and analyzed by the linear ion trap using 1e4 AGC target after HCD fragmentation at 30% collision energy with 50ms maximum injection time. The MS3 scans (100–500 m/z) were acquired in the Orbitrap at 50,000 resolution, with a 1e5 AGC, 2 m/z MS2 isolation window, at 105ms maximum injection time after HCD fragmentation with a normalized energy of 65%. Precursor ions were selected in 400–2000 m/z mass range with mass exclusion width of 5–18 m/z. Polysiloxane 371.10124 m/z was used as the lock mass.

The raw mass spectrometry data was searched with MaxQuant (1.6.6.0). Search parameters were as follows: specific tryptic digestion, up to 2 missed cleavages, a static carbamidomethyl cysteine modification, variable protein N-term acetylation, and variable phospho(STY) as well as methionine oxidation using the human UniProtKB/Swiss-Prot sequence database (Downloaded Feb 1, 2017). MaxQuant data was deposited to PRIDE/Proteome Xchange.

### Fractionation

Following treatment, MDA-MB-231 cells were harvested, and using NE-PER^™^ Nuclear and Cytoplasmic Extraction Reagents (ThermoScientific), nuclear fractionation was performed according to manufacturer’s instructions.

### Cell proliferation assays

Cells were seeded in replicates of 6, at a density of 2,500 cells/well in 96 well plates and allowed to attach overnight. Next day, media were changed to assay media, supplemented with or without agents as indicated. Cell proliferation was assayed after 6 days using the supravital dye neutral red (NR) incorporation. The medium was removed, 0.2 ml of medium containing 0.04 mg/ml NR was added per well, and incubation was continued for 30 min at 37°C. Cells were then rapidly washed and fixed with a 0.2-ml solution of 0.5% formalin, 1% CaCl_2_ (v/v), and the NR incorporated into the viable cells was released into the supernatant with a 0.2-ml solution of 1% acetic acid, 50% ethanol. Absorbance was recorded at 540 nm with a microtiter plate spectrophotometer. Experiments were performed a minimum of three times. Cytotoxicity graphic data were presented as the mean percentages of control ± standard deviation (STDEV).

### Wound healing assay

Cells were seeded in 6-well plates in complete DMEM media, supplemented with 10% FBS and grown to confluency in monolayer overnight. Wound/scratch was created along the diameter of each well using a 200μl pipette tip. Cell debris were removed by washing once with PBS, followed by addition of fresh DMEM media supplemented with 1% FBS, with or without agents as indicated. Cell imaging and migration were measured using Keyence BZX-800 microscope. Data was analyzed and plotted using Excel. Experiments were performed a minimum of three times.

### Boyden chamber assay

Cells were cultured in media without serum and treated with either XCT-790, tamoxifen and/or U0126, as indicated. Nineteen hours later, 1 × 10^5^ cells per well were plated on tissue culture inserts with 8.0-μm pores. The inserts were incubated with serum-free media containing 10 μM XCT-790, 100 nM tamoxifen or 10 μM U0126, alone or in combination. Complete media were added to the lower chamber and cells allowed to migrate for 15 hours. After 15 hours, cells remaining on the upper side of the membrane were scraped off, and the cells that had migrated to the lower side of the membrane were fixed in 4% paraformaldehyde. The insert membranes were removed, stained, and mounted on coverslips using DAPI Fluoromount. Images were collected at 10× magnification using Keyence BZX-800 microscope. Nuclei were counted manually, and data were analyzed using two-tailed Student’s *t* test and plotted using Excel. Experiments were performed a minimum of three times.

### Microscopy

Cells were imaged under phase contrast lens using the Keyence BZX-800 microscope with a 20x objective.

### Statistical analysis

All experiments were repeated at least thrice. Data was analyzed using Excel and significance of data was determined using t-tests, where * represents *P* values <0.05, ** represents *P* values <0.01, and *** represents *P* values <0.001.

## Results

**Fig 1:**
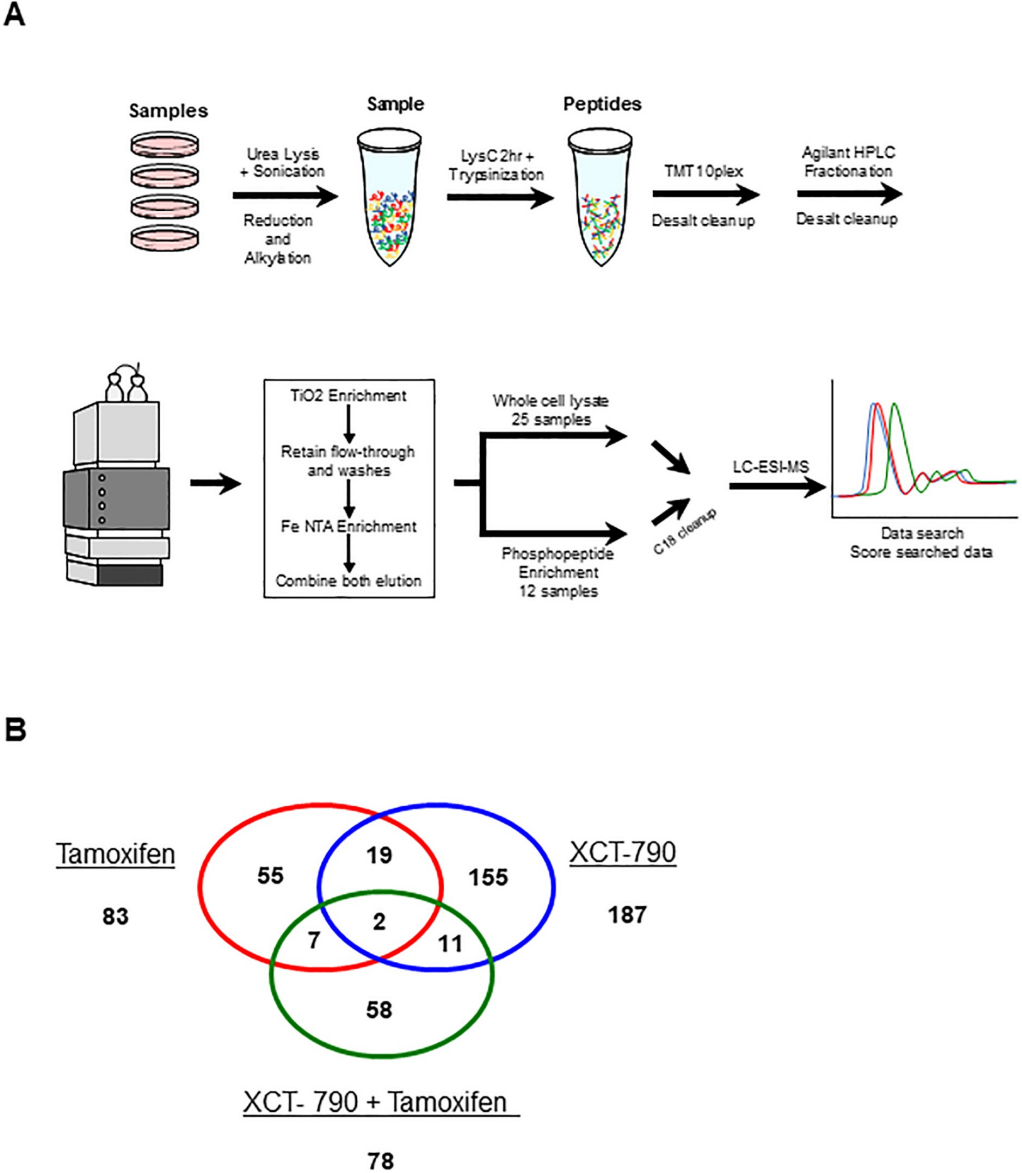
Phosphoproteomic analysis of XCT-790 and tamoxifen treated MDA-MB-231 cells. (A) Steps of the work flow chart of samples treated for the phosphroproteomic analysis. (B) Outcome of number of identified unique phosphorylated proteins in each group.

To identify common targets that are regulated by both tamoxifen as well as ERRα inverse agonist XCT-790 in TNBC, phosphoproteomic analysis was performed on MDA-MB-231 cells (Fig 1A, S1 Fig and S1 Table). Bioinformatic analysis identified 307 unique targets with statistically significant phosphorylation (*P*<0.05) changes (Fig 1B and S2 Table). Further analysis of the data identified 19 unique phosphosites that were changed in both tamoxifen and XCT-790 treated cells (Table 1). A specific direct target which was of particular interest to us was MAPK1, whose phosphorylation was upregulated on tyrosine 187 (also known as p-ERK Y204/187) upon treatment with tamoxifen as well as with XCT-790. MAPK1 is a member of the Ras/Raf/MEK/ERK signaling pathway that regulates cell cycle progression as well as apoptosis and warrants further study as an important player in TNBC progression.

**Table 1:** Significant phosphoproteomic changes inXCT-790 as well as tamoxifen treated samples. A table of statistically significant hits from phosphoproteomic analysis of cells treated with XCT-790 as well as tamoxifen.

Protein	Protein names	Gene names	Position
Q03252	Lamin-B2	LMNB2	420
Q9Y2U5	Mitogen-activated protein kinase kinase kinase 2	MAP3K2	239
Q08170	Serine/arginine-rich splicing factor 4	SRSF4	431
Q9Y2D5	A-kinase anchor protein 2	AKAP2	720
Q3KQU3-2	MAP7 domain-containing protein 1	MAP7D1	796
Q9BWF3-3	RNA-binding protein 4	RBM4	86
Q9UKV3-5	Apoptotic chromatin condensation inducer in the nucleus	ACIN1	408
Q8WZ73-3	E3 ubiquitin-protein ligase rififylin	RFFL	212
Q5JSH3-2	WD repeat-containing protein 44	WDR44	163
Q13547	Histone deacetylase 1	HDAC1	421
Q96RT1-7	Protein LAP2	ERBB2IP	1015
P42858	Huntingtin	HTT	432
Q13158	FAS-associated death domain protein	FADD	194
Q9NXH8	Torsin-4A	TOR4A	63
Q8IYB3-2	Serine/arginine repetitive matrix protein 1	SRRM1	595
Q9UQ35	Serine/arginine repetitive matrix protein 2	SRRM2	1014
Q8NCF5	NFATC2-interacting protein	NFATC2IP	204
Q15366-7	Poly(rC)-binding protein 2	PCBP2	317
P28482-2	Mitogen-activated protein kinase 1	MAPK1	187

**Fig 2:**
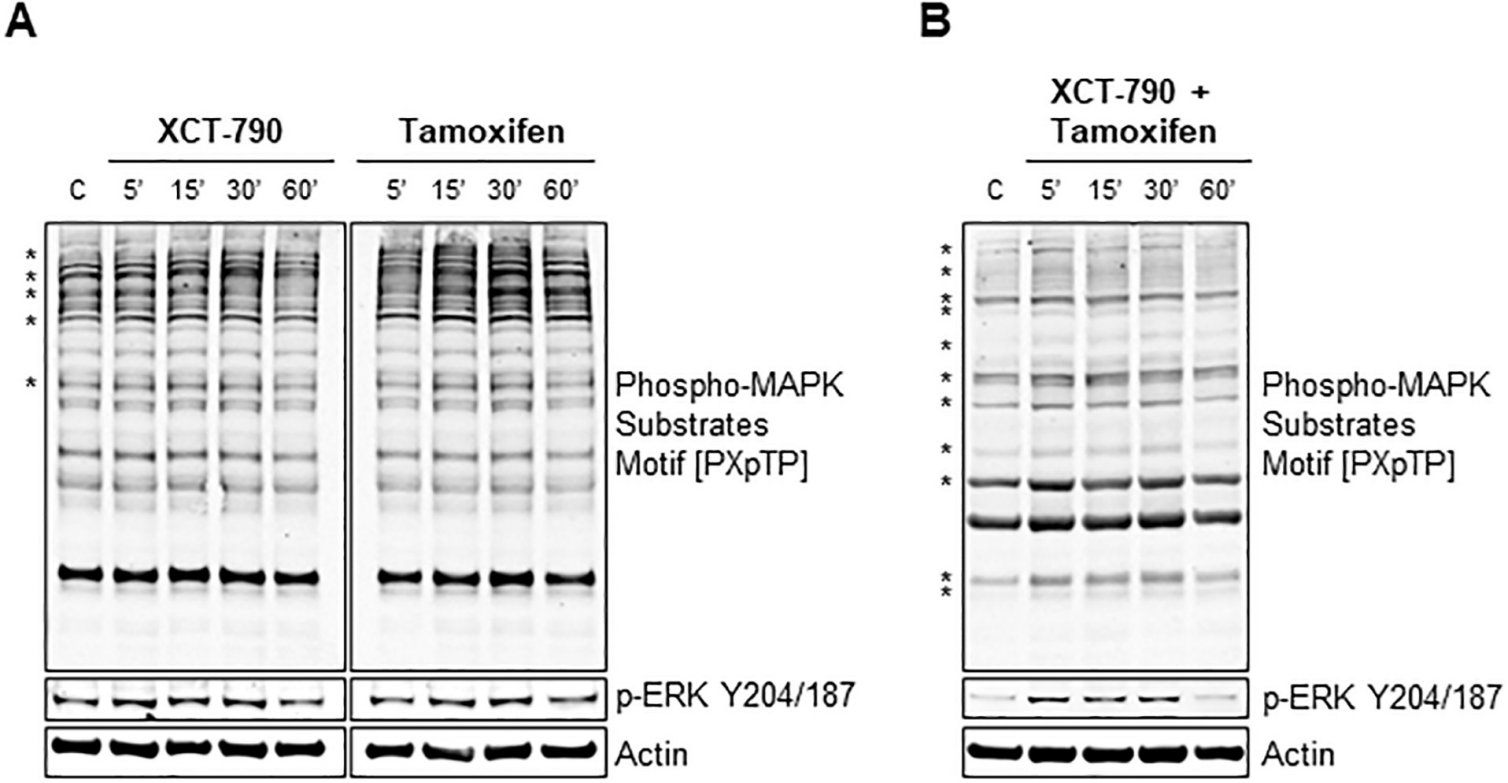
XCT-790 and tamoxifen treatments regulate signaling of MAPK pathway. (A) MDA-MB-231 cells were grown in starvation media for 24 hrs and treated with either 10 μM XCT-790 or 100 nM tamoxifen for 5, 15, 30 or 60 minutes. Cells were lysed as described in “*Materials and Methods”* and indicated proteins were detected by immunoblot. (B) MDA-MB-231 cells were grown in starvation media for 24 hrs and treated with 10 μM XCT-790 and 100 nM tamoxifen for 5, 15, 30 or 60 minutes. Cells were lysed as described in “*Materials and Methods”* and indicated proteins were detected by immunoblot. * indicates phosphorylation changes in [PXpTP] motif as compared to control. ‘C’ is untreated control.

**Fig 3:**
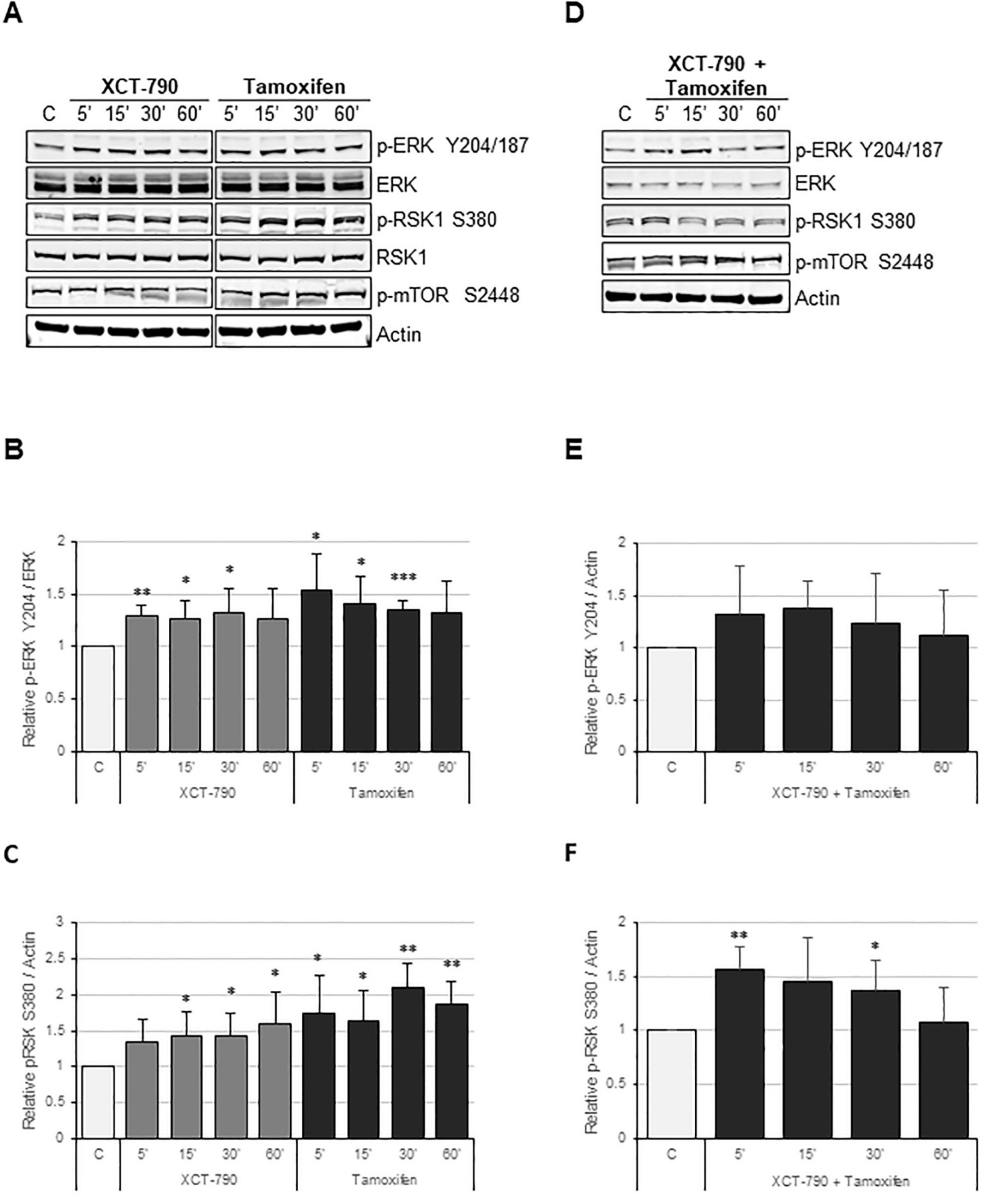
XCT-790 and tamoxifen treatments upregulate phosphorylation of p-ERK on Y204/187. (A) MDA-MB-231 cells were grown in starvation media for 24 hrs and treated with either 10 μM XCT-790 or 100 nM tamoxifen for 5, 15, 30 or 60 minutes. Cells were lysed as described in “*Materials and Methods”* and indicated proteins were detected by immunoblot. (B) Quantification of p-ERK Y204/187 protein levels normalized to total ERK signal from *‘A’*. (C) Quantification of p-RSK1 S380 protein levels normalized to actin signal from *‘A’*. (D) MDA-MB-231 cells were grown in starvation media for 24 hrs and treated with 10 μM XCT-790 and 100nM tamoxifen for 5, 15, 30 or 60 minutes. Cells were lysed as described in “*Materials and Methods”* and indicated proteins were detected by immunoblot. (E) Quantification of p-ERK Y204/187 protein levels normalized to actin signal from *‘D’*. (F) Quantification of p-RSK1 S380 protein levels normalized to actin signal from *‘D’*. * represents *P*<0.05, ** represents *P*<0.01 and *** represents *P*<0.001.

To validate phosphoproteomic findings, MDA-MB-231 cells were grown in serum-free media for 24 hrs and treated with 100 nM tamoxifen and 10 μM XCT-790, alone or in combination as indicated and probed with an antibody that recognizes proteins that are phosphorylated at the threonine within the PXpTP motif, also known as the phospho-MAPK substrate motif (Fig 2). As compared to the untreated control, phosphorylation changes in phospho-MAPK substrate motif [PXpTP] are readily seen and are indicated by asterisks. In tamoxifen or XCT-790 treated samples, phospho-MAPK substrate changes are seen by 15–30 min post treatment (Fig 2A) and in samples treated with combination of tamoxifen and XCT-790, phospho-MAPK substrate changes are pronounced as early as 5 min post treatment (Fig 2B). Counterstaining of the membrane with an antibody specific for the phosphorylation of MAPK1 on tyrosine 187 (p-ERK Y204/187) showed upregulation in phosphorylation upon treatment with XCT-790 and tamoxifen, alone or in combination. Therefore, western blot analysis validated the phosphoproteomic findings and further showed that XCT-790 and tamoxifen treatments regulate MAPK signaling pathway globally in TNBC. To further validate phosphoproteomic findings and explore the relationship between ERRα, tamoxifen, and ERK in the context of TNBC, MDA-MB-231 cells were grown in serum-free media for 24 hrs and treated with tamoxifen and XCT-790, alone or in combination over a time course of 5, 15, 30 and 60 min (Fig 3). Immunoblotting verified that ERRα inhibition by XCT-790 caused statistically significant upregulation of p-ERK Y204/187 as early as 5, 15 and 30 min post treatment (Fig 3A and 3B). Likewise, tamoxifen treatment caused statistically significant upregulation of p-ERK Y204/187 as early as 5, 15 and 30 min post treatment and similar results were observed in the combination of XCT-790 and tamoxifen treated samples (Fig 3D and 3E). Importantly, statistically significant upregulation of p-RSK1 S380, a direct downstream target of ERK, was observed in XCT-790 and tamoxifen treated samples, alone or in combination (Fig 3A and 3C). This finding validates phosphoproteomic’s findings and indicates that MEK/ERK/RSK signaling pathway is regulated by ERRα and tamoxifen in TNBC. Additionally, this effect is specific to the MAPK signaling pathway, as XCT-790 or tamoxifen treatment had no impact on activation of mTOR on S2448 (Fig 3A and 3D). Mammalian target of rapamycin (mTOR) is a parallel signaling pathway that is activated by growth factors and is often hyperactivated in cancer, including breast cancer [34]. These results indicate that ERRα and tamoxifen regulate p-ERK and that this effect is direct and specific to the MAPK signaling pathway.

**Fig 4:**
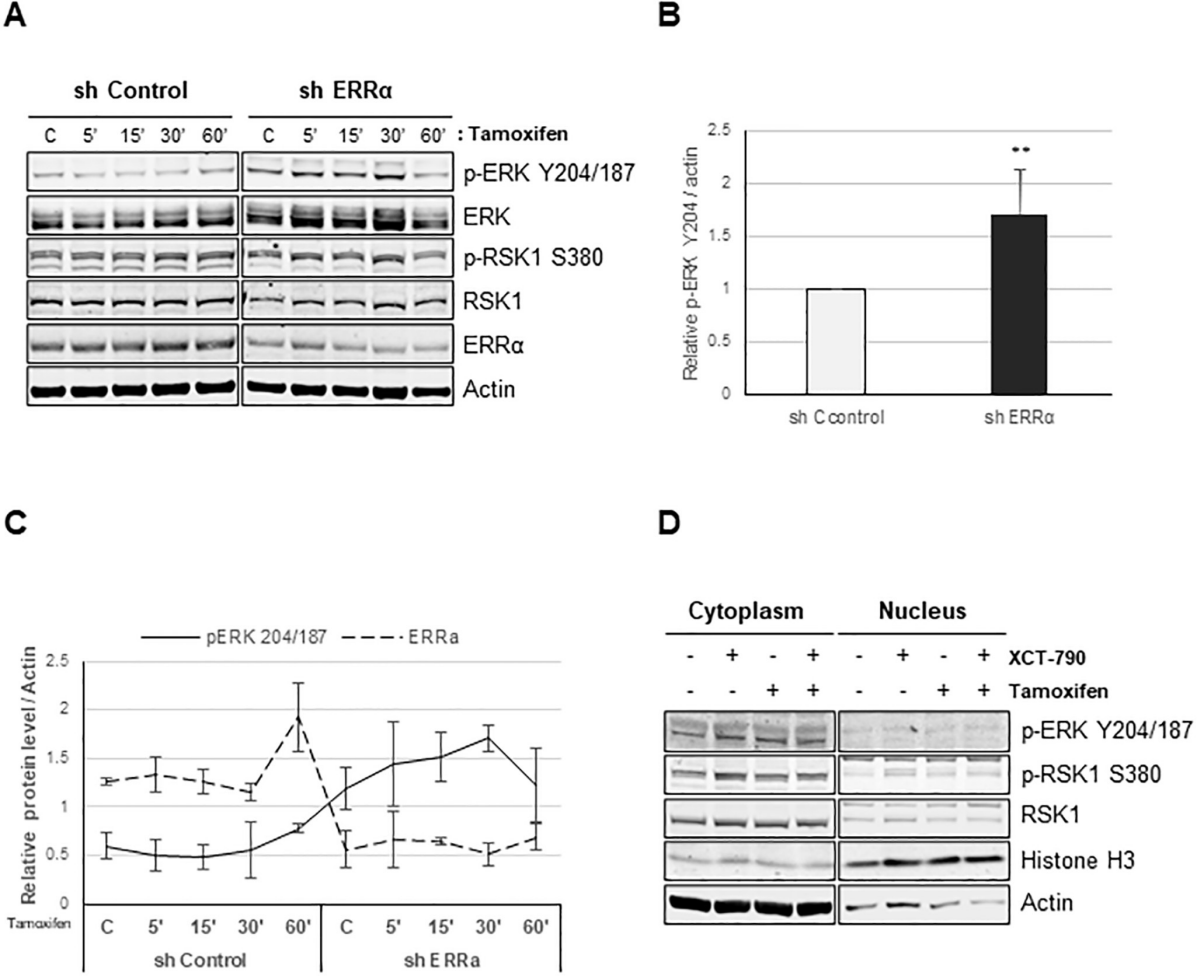
Tamoxifen potentiates phosphorylation of ERK on Y204/187 in the absence of ERRα. (A) sh Control and sh ERRα cells were treated with 100 nM of tamoxifen for 5, 15, 30, or 60 minutes. Cells were lysed as described in *“Materials and Methods”* and the indicated proteins were detected by immunoblot. (B) Relative levels of p-ERK Y204/187 normalized to actin, ** represents *P*<0.01. (C) Relative levels of p-ERK Y204/187 and ERRα, normalized to actin from *‘A’*. (D) MDA-MB 231 cells were treated with 10 μM XCT-790 and/or 100 nM tamoxifen for 30 minutes followed by lysis with NE-PER Nuclear Cytoplasmic extraction kit as described in *‘Materials and Methods’* and lysates from nuclear and cytoplasmic extractions were immunoblotted with the indicated antibodies.

In an effort to define the relationship between ERRα levels and tamoxifen response, as well as to explore the role of ERRα in the phosphorylation of ERK, MDA-MB-231 cells with stable knockdown of ERRα (shERRα), were grown in serum-free media for 24 hrs and treated with tamoxifen for 5, 15, 30 and 60 min (Fig 4). As compared to control, MDA-MB-231 cells with reduced ERRα expression showed 2-fold upregulation of phosphorylation of p-ERK on Y204/187 (Fig 4A and 4B). This validates our previous findings and indicates that the effect of XCT-790 on ERK phosphorylation is due to its inhibition of ERRα. Furthermore, treatment of either MDA-MB-231 cells or cells with reduced expression of ERRα, showed that there is an inverse relationship between ERRα expression and phosphorylation of ERK on Y204/187, such that high ERRα expression in MDA-MB-231 cells is correlated with low p-ERK Y204/187 expression and reduced ERRα expression in sh-ERRα cells is correlated with increased phosphorylation of p-ERK on Y204/187 (Fig 4A and 4C). This finding further validates phosphoproteomic data and shows that inhibition of ERRα by XCT-790 as well as use of tamoxifen activate ERK signaling, and this has tremendously important implications for TNBC patients as we have identified a signaling pathway and a specific molecular target whose inhibition can be used therapeutically.

Since ERK is known to have both cytoplasmic as well nuclear functions, it is important to determine subcellular localization of p-ERK and its function. MDA-MB-231 cells were grown in serum-free media for 24 hrs, treated with tamoxifen and XCT-790, alone or in combination for 30 min, followed by preparation of nuclear and cytoplasmic fractions. As seen in Fig 4D, tamoxifen and XCT-790 treatment, alone or in combination, upregulated phosphorylation of ERK on Y204/187 specifically in the cytoplasm, and no p-ERK expression was detected in the nuclear fraction at this time point. Consistent with ERK’s cytoplasmic function, its downstream target RSK1 was also phosphorylated on S380 upon tamoxifen as well as XCT-790 treatment, alone or in combination, and this was observed only in the cytoplasmic fraction and not in the nuclear fraction. The presence of histone H3 mainly in the nuclear fraction and presence of actin mainly in the cytoplasmic fraction served as technical control (Fig 4D).

**Fig 5:**
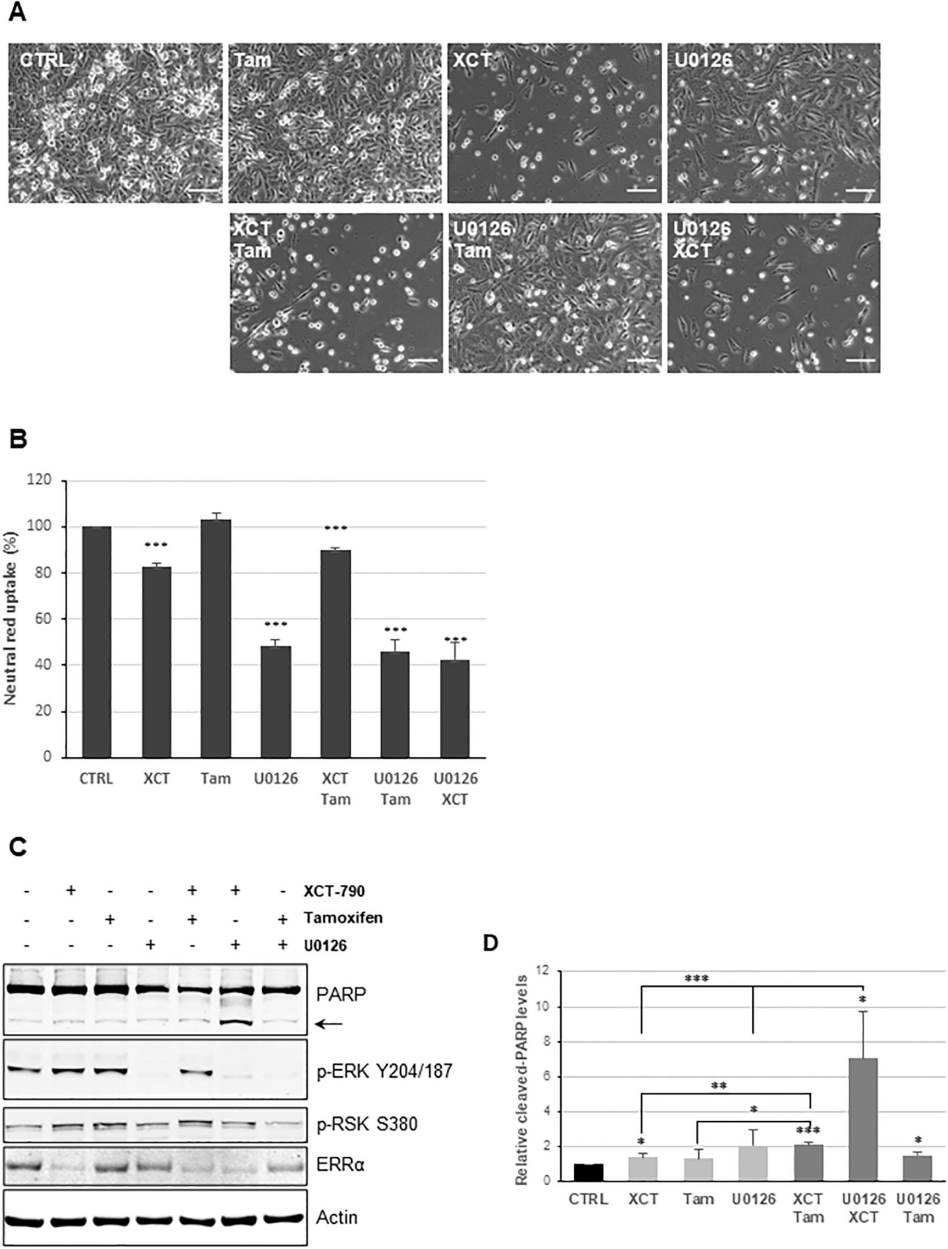
Inhibition of ERRα potentiates apoptotic effects of U0126 in TNBC cells. (A) MDA-MB-231 cells were serum starved for 24 hrs and treated with 10 μM XCT-790, 100 nM tamoxifen, or 10 μM U0126, alone or in combination for 48 hrs. Cells were imaged under 20x magnification using BZX-800 microscope from Keyence. Scale bar represents 100 μm. (B) MDA-MB-231 cells were seeded in 96-well plate in complete DMEM media, supplemented with 10% FBS and treated with 10 μM XCT-790, 100 nM tamoxifen, or 10 μM U0126, alone or in combination for 6 days and cell viability was measured using Neutral Red cytotoxicity assay as described in *“Materials and Methods*”. (C) MDA-MB-231 cells were serum starved for 24 hrs and treated with 10 μM XCT-790, 100 nM tamoxifen, or 10 μM U0126, alone or in combination for 24 hrs as indicated. Cells were lysed as described in *“Materials and Methods”* and immunoblotted for indicated proteins. Arrow indicates cleaved PARP fragment. (D) Quantification of cleaved PARP fragment from “*B”* normalized to actin. * represents *P*<0.05, ** represents *P*<0.01 and *** represents *P*<0.001.

Since XCT-790 and tamoxifen treatments upregulate phosphorylation of ERK, this paves the way for investigating the use of MAPK inhibitors as a therapeutic strategy for TNBC patients. To that end, we investigated the effectiveness of U0126, a MEK1/2 inhibitor, in the context of TNBC cells. MDA-MB 231 cells were grown in serum free media and treated with 10 μM XCT-790, 100 nM tamoxifen and 10 μM U0126, alone or in combination (Fig 5). Following 48 hrs of treatment, cells were imaged with Keyence BZX-800 microscope under phase contrast (Fig 5A). Tamoxifen treatment alone did not have an effect on cell growth and density, however U0126 treatment and to a greater extent XCT-790 treatment showed a significant reduction in cell growth and density. Additionally, combination of tamoxifen together with XCT-790 as well as U0126 together with XCT-790 had a drastic and pronounced effect on cell density with very few cells attached. The attached cells were not healthy and appeared spindle-like or rounded up and majority of cells were floating dead cells. To quantify cell viability, relative uptake of neutral red dye by the lysosomes of live cells was measured. MDA-MB-231 cells were seeded in a 96-well plate in full serum media, treated with 10 μM XCT-790, 100 nM tamoxifen and 10 μM U0126, alone or in combination and neutral red assay was performed 6 days post treatment (Fig 5B). Consistent with previous observation, cells treated with XCT-790 had approximately 20% reduction in cell viability, while treatment of cells with U0126 alone or in combination with tamoxifen or XCT-790 had greater than 50% reduction in cell viability.

To further investigate whether the effect on cell viability is due to cell death, rather than inhibition of cell growth, expression level of cleaved-PARP fragment, a marker of apoptotic cell death was investigated (Fig 5C and 5D). MDA-MB-231 cells were grown in serum free media for 24 hrs and treated with 10 μM XCT-790, 100 nM tamoxifen and 10 μM U0126, alone or in combination for 24 hrs. As expected, U0126 treatment blocked activation of ERK signaling as seen via reduction of p-ERK Y204/187 and XCT-790 treatment reduced ERRα protein levels. Additionally, p-ERK Y204/187 and p-RSK1 S380 levels were upregulated upon treatment with XCT-790 and tamoxifen, validating previous data (Fig 5C). Most importantly, we observed statistically significant upregulation in apoptosis as measured by quantification of cleaved-PARP fragment (Fig 5D), indicated by the arrow (Fig 5C). The most notable increase in cleaved PARP levels was observed in samples treated with a combination of U0126 and XCT-790, which exhibited the greatest level of cleaved PARP when compared to cells treated with either U0126 or XCT-790, alone. This finding is very exciting as it indicates that the use of MEK inhibitors together with inhibition of ERRα is effective at inducing apoptosis in TNBC cells and is a first indication of designing a targeted therapy for treatment of TNBC.

**Fig 6:**
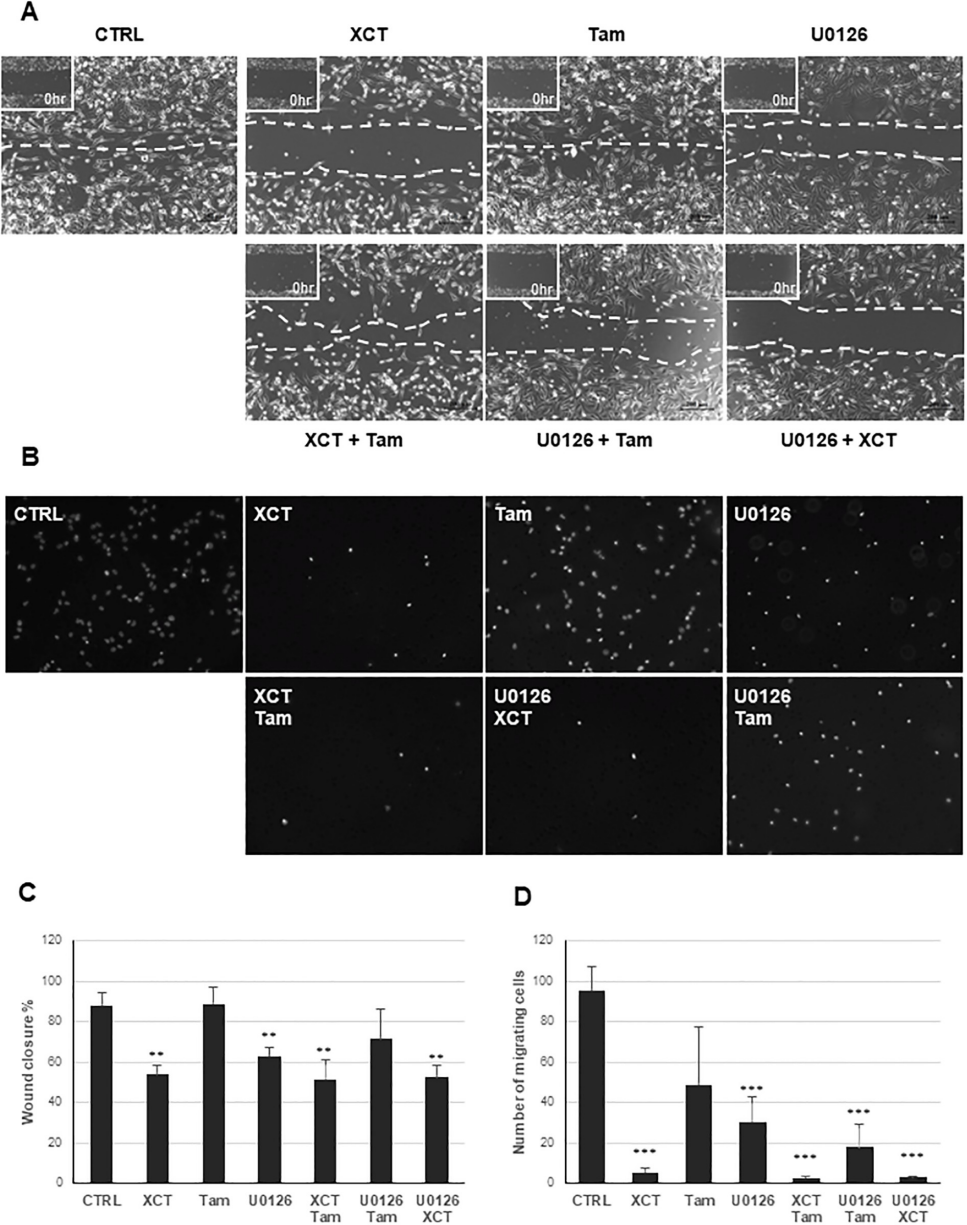
Inhibition of ERRα together with U0126 prevents cell migration and invasion in TNBC cells. (A) MDA-MB-231 cells were seeded in a 6-well plate in complete DMEM media and allowed to attach overnight. The following day cells were placed in reduced (1%) serum media and following 8–10 hrs of pretreatment with 10rμM XCT-790, 100nM tamoxifen, or 10rμM U0126, alone or in combination and wound/scratch was generated. Wound closure was measured 17 hrs post generation. (B) Cell migration/Boyden chamber assay was performed as described in *“Materials and Methods”*. Representative images of MDA-MB-231 cells stained with 4′,6-diamidino-2-phenylindole (DAPI) following 15 hrs migration assay are shown. (C) Quantification of the Wound Healing assay from *(A)* was performed and graphed using paired Student’s t-test. * represents P<0.05. ** represents P<0.01. (D) Histogram representing the number of cells migrated relative to untreated control. * represents *P*<0.05, ** represents *P*<0.01 and *** represents *P*<0.001.

We subsequently wanted to examine whether such therapy is effective at inhibition migration and invasion of TNBC cells. To test the effect on cell motility, wound-healing assay was performed and quantified using MDA-MB-231 cells (Fig 6A and 6C). Though tamoxifen treatment alone did not have an effect on cell migration, treatment with either XCT-790 or U0126 had a statistically significant effect on inhibiting cell migration and this effect was also seen in combination therapy of XCT-790 with either tamoxifen or U0126. To test the effect on cell invasion, trans-well migration assay was performed and quantified using MDA-MB-231 cells (Fig 6B and 6D). Similar trend was observed in the trans-well migration assay as in the wound-healing assay. In particular, both XCT-790 and U0126 treatments had statistically significant inhibition on cell migration and this effect was maintained in all three combinations of treatments.

**Fig 7:**
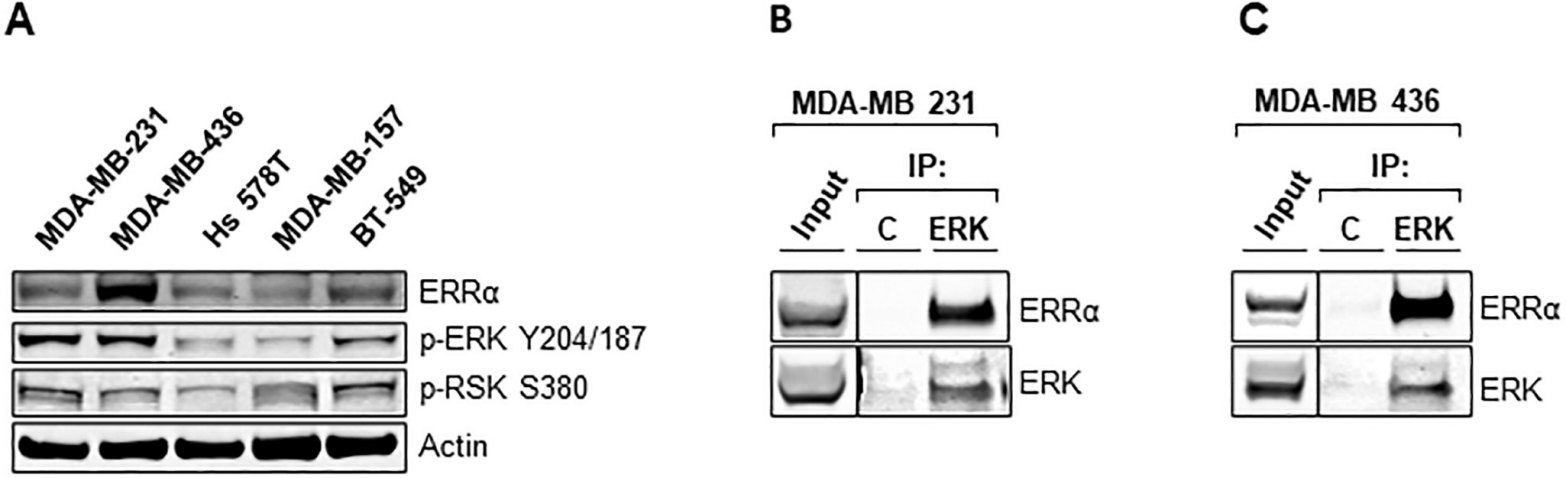
ERRα directly binds to ERK. (A) The represented TNBC cell lines were lysed as described in *“Materials and Methods”* and immunoblotted for the indicated proteins. (B) MDA-MB 231 cells were lysed and immunoprecipitated as described in *“Materials and Methods”* and proteins were detected by immunoblot. (C) MDA-MB 436 cells were lysed and immunoprecipitated as described in *“Materials and Methods”* and proteins were detected by immunoblot.

To verify that such therapy is beneficial to treatment of TNBC and is not unique to MDA-MB-231 cells, expression levels of ERRα and active signaling of MEK/ERK/RSK pathway were investigated in a panel of TNBC cells (Fig 7A). As expected, all of the tested TNBC cells express ERRα, but they also show active MEK/ERK/RSK signaling as indicated by phosphorylation of ERK on Y204/187 and RSK on S380. To identify the mechanism of XCT-790 action on ERK signaling, we wanted to see whether ERRα and ERK interact using co-immunoprecipitation assay using whole cell lysates. We confirmed that ERK and ERRα interact using MDA-MB 231 (Fig 7B) as well as high- ERRα expressing MDA-MB 436 (Fig 7C) cell lines.

## Discussion

Despite significant strides that have been made in the development of treatments for hormone receptor positive breast cancer, progress in the treatment of TNBC has lagged behind, leaving patients with few treatment options. Results from previous studies identified a subset of patients with TNBC who have high ERRα expression and respond to tamoxifen treatment [25]. This finding prompted us to further investigate the role of ERRα in conferring tamoxifen sensitivity in TNBC tumors and to discover signaling pathways responsible for ERRα induced tamoxifen sensitivity, with the aim of identifying specific druggable target for the treatment of TNBC. In our study, we discovered that the MAPK signaling pathway is regulated by tamoxifen as well as ERRα and that attenuation of this signaling pathway may be a promising therapeutic strategy for the treatment of TNBC. Though the mechanism of tamoxifen action in TNBC cells is not clear, since ERRα and ERα can regulate a subset of common target genes, specifically ones with high relevance to breast tumor biology [35], it is possible that tamoxifen might have a similar mechanism of action on ERRα as it does on ERα. Of the targets we identified through phosphoproteomic analysis, MAPK1 phosphorylation at Y204/187 was particularly interesting as the MAPK pathway has been implicated in many processes associated with cancer progression including, tumor proliferation, invasion, metastasis, migration, and apoptosis [36, 37]. MAPK1 is part of a kinase signaling cascade that begins extracellularly with the EGFR receptor and cascades through a series of kinases including Ras, Raf, MEK, and ERK 1/2 consecutively [38]. ERK 2 is also known as MAPK1 and is a serine threonine kinase [39]. The identification of ERK as a target of ERRα and tamoxifen identifies an important target whose modulation should be further explored clinically as a therapy in TNBC.

Further validation of the phosphoproteomic result with immunoblotting confirmed that XCT-790 as well as tamoxifen treatment led to an increase in ERK phosphorylation on Y204/187 as well as phosphorylation of the downstream kinase cascade. Our results show that ERRa directly binds to MAPK1, and through this interaction leads to modulation of MAPK1 phosphorylation on Y204/187 as well as downstream signaling. Since MAPK activation is known to support cellular survival, we hypothesized that this activation might be a compensatory cellular signal in response to treatment. Such compensatory responses have been previously described and have been implicated in the development of drug resistance [40]. Crosstalk from other pathways such as PI3K/Akt/mTOR or activation feedback loops within the Raf/MEK/ERK signaling pathway have been previously implicated in pro-survival compensatory mechanisms and drug resistance, thus encouraging further research into the use of drug combinations that act on multiple targets and disrupt the molecular compensatory mechanisms [37, 41, 42]. Though activation of Raf/MEK/ERK signaling is associated with tumor progression, it also identified an ERRα dependent target whose inhibition can be explored clinically for treatment of TNBC tumors.

Strategies to curb tumor progression through the inhibition of the MAPK pathway have been previously described and are currently used in the treatment of colon cancer and melanoma [43–53]. In breast cancer, preclinical studies described reduced tumor volume in xenograft models, induced cell cycle arrest, and increased apoptosis with the attenuation of the MAPK pathway [54, 55]. Though these results have yet to be validated in clinical trials, inhibition of EGFR in breast cancer was tested clinically due to evidence of EGFR overexpression in nearly half of TNBCs [43, 44]. Results from those trials were limited showing benefit only in certain subgroups of patients and clinical trials exploring the use of MEK inhibitors in breast cancers are currently underway. In our work, we showed that a combination of ERRα inverse agonist and MEK inhibition leads to statistically significant reduction in cell proliferation, upregulation of apoptosis as well as inhibition of cell migration and invasion.

TNBC is a highly aggressive and metastatic form of breast cancer with a poor prognosis and poor patient outcome. To date, we do not have a clear understanding of molecular pathways that modulate cancer progression and therefore no targeted treatment therapies exist for TNBC patients. What is exciting and important is that we, for the first time, present data that show a direct link between ERRα and ERK signaling pathway. ERRα is a transcription factor that is highly expressed in TNBC and is associated with a more aggressive cancer and a poor outcome. We have shown that ERRα directly binds to ERK and modulates its phosphorylation on Y204/187 as well as downstream pathway activation, indicating that inhibitors of the MEK/ERK signaling pathway should be considered therapeutically for treatment of TNBC. As a proof of this concept, we showed that the use of MEK1/2 inhibitor alone or in combination with XCT-790 induces apoptosis as well as inhibits migration and invasion in TNBC cells. This is a highly exciting finding for TNBC patients and indicates that further investigation into clinical use of MEK inhibitors should be done for treatment of TNBC.

## Supporting information

S1 Fig Principal component analysis (PCA). Principal component analysis of protein abundance of MDA-MB 231 cells treated with tamoxifen, XCT-790 or tamoxifen plus XCT-790.
(PDF)

S1 Table All phosphoproteomic targets. (XLSX)

S2 Table Phosphotargets with statistically significant changes in phosphorylation in at least one treatment condition (*P*<0.05 in green). (XLSX)

S1 Raw images (PDF)
