# Supplementary figures and images for: Inhibition of ERK signaling for treatment of ERRα positive TNBC
Source: PLoS One. 2023 May 10;18(5):e0283047. doi: 10.1371/journal.pone.0283047 (PMC10171695; doi:10.1371/journal.pone.0283047)

# Principal Component Analysis (PCA) – Phospho

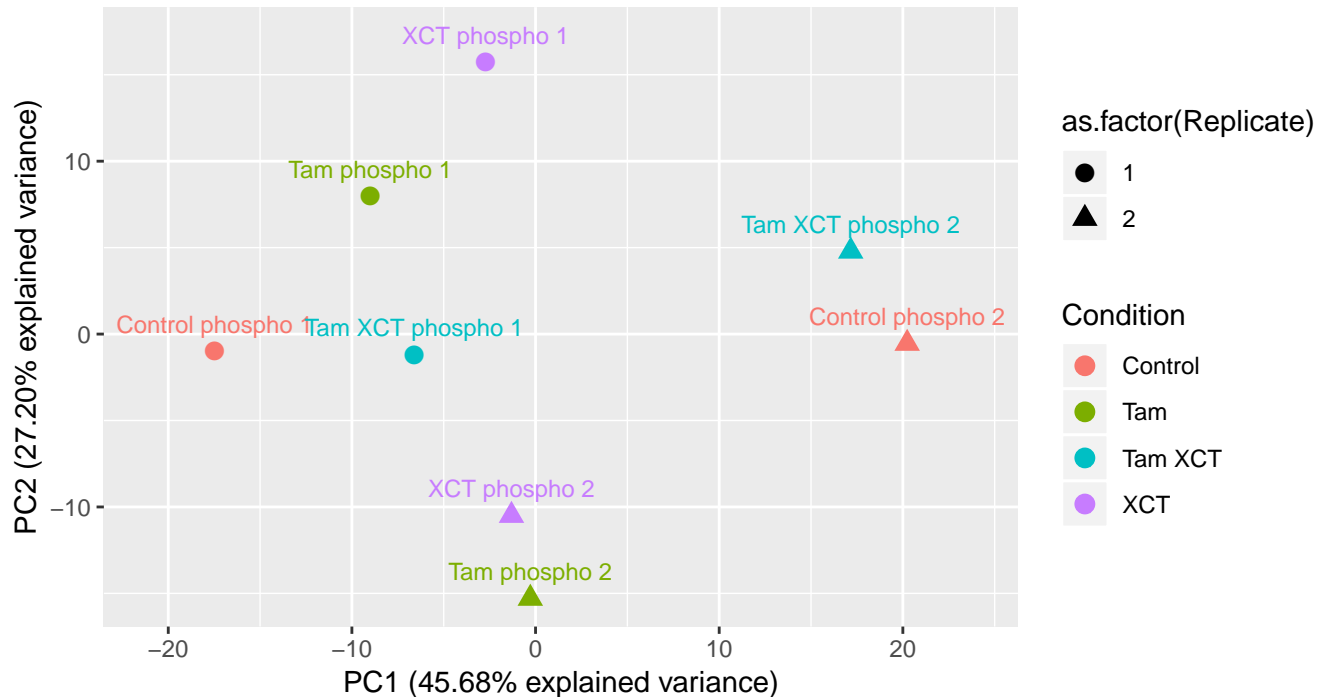

Supplement: S1 Fig — Principal component analysis of protein abundance of MDA-MB 231 cells treated with tamoxifen, XCT-790 or tamoxifen plus XCT-790. (PDF) [file pone.0283047.s001.pdf]
