# Supplementary material for: Inhibition of ERK signaling for treatment of ERRα positive TNBC
Source: PLoS One. 2023 May 10;18(5):e0283047. doi: 10.1371/journal.pone.0283047 (PMC10171695; doi:10.1371/journal.pone.0283047)

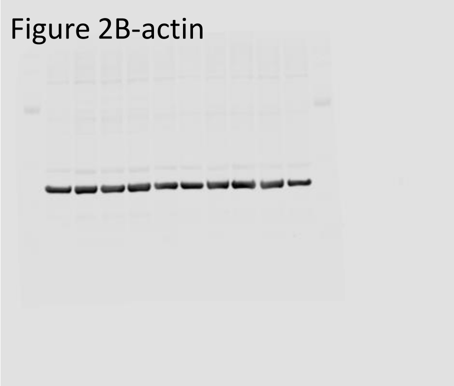
Figure 2B-actin

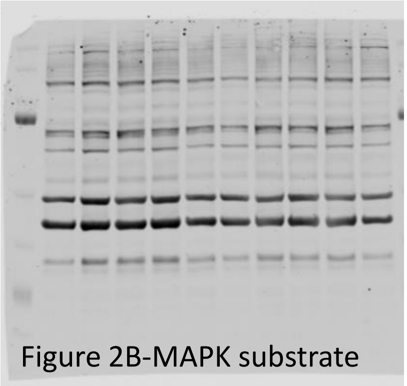
Figure 2B-MAPK substrate

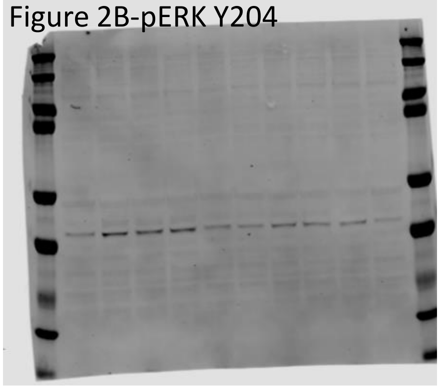
Figure 2B-pERK Y204

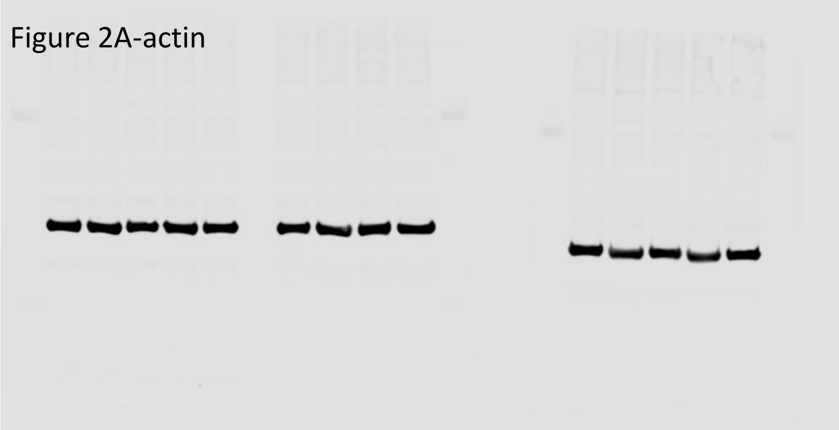
Figure 2A-actin

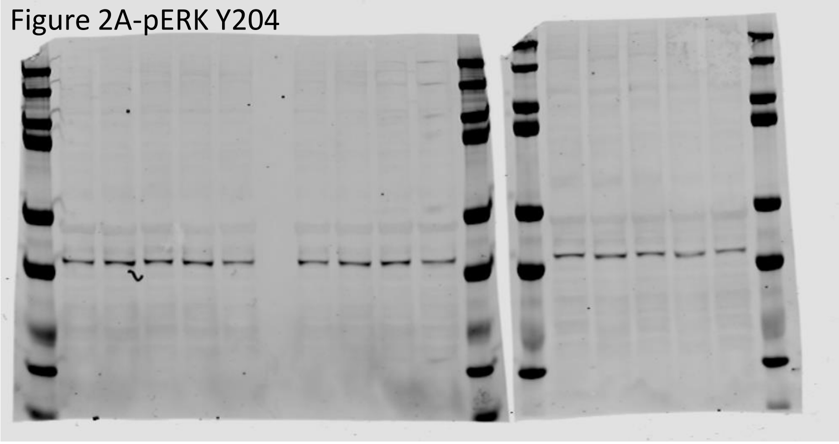
Figure 2A-pERK Y204

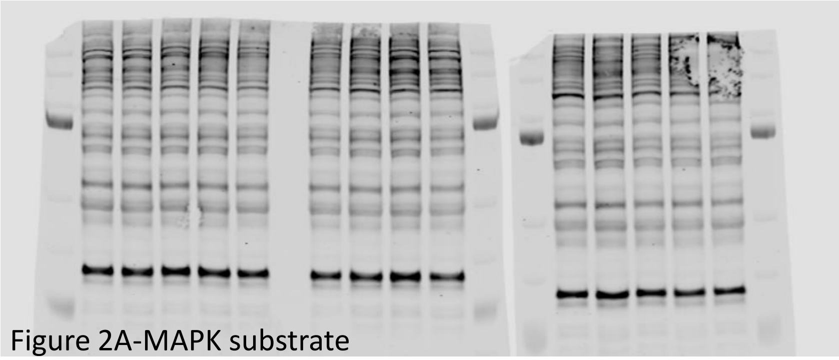
Figure 2A-MAPK substrate

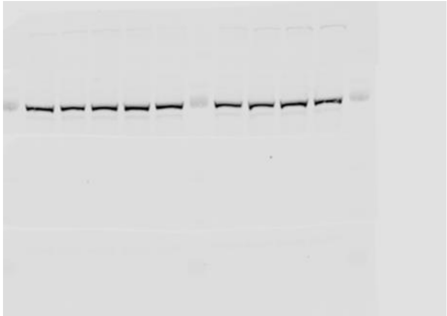

Figure 3A-RSK

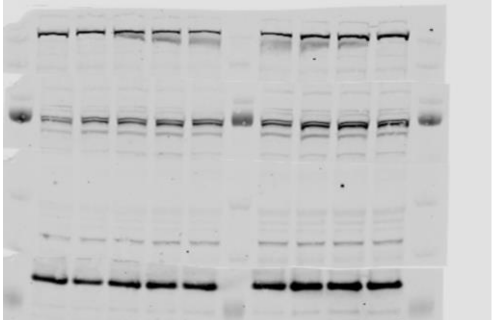

Figure 3A-pmTOR s2448_pRSK 380

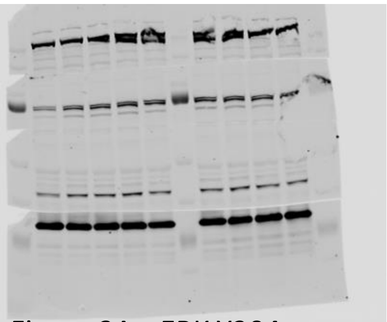

Figure 3A-pERK Y204

Figure 3D-pmTOR s2448

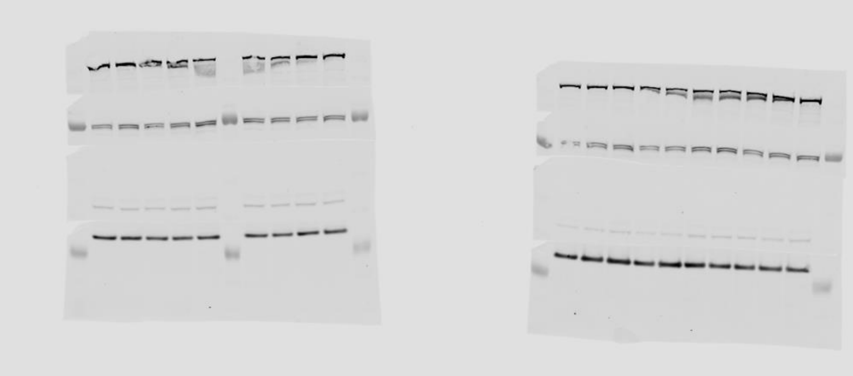

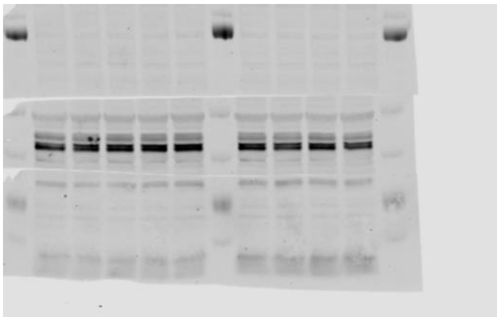

Figure 3A-ERK

Figure 3D-actin

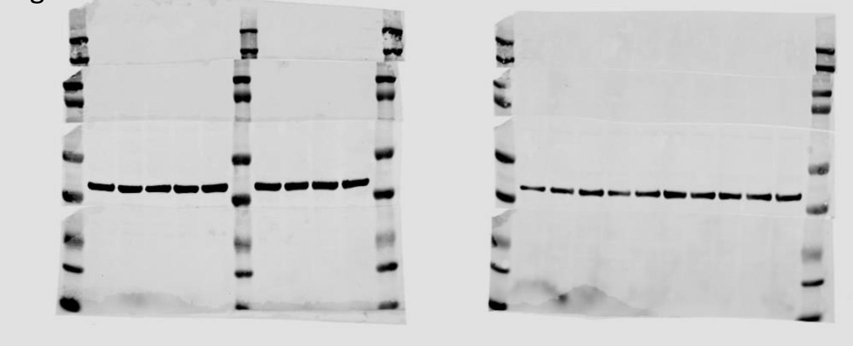

Figure 3A-actin

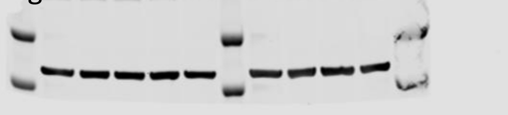

Figure 3D-pERK Y204

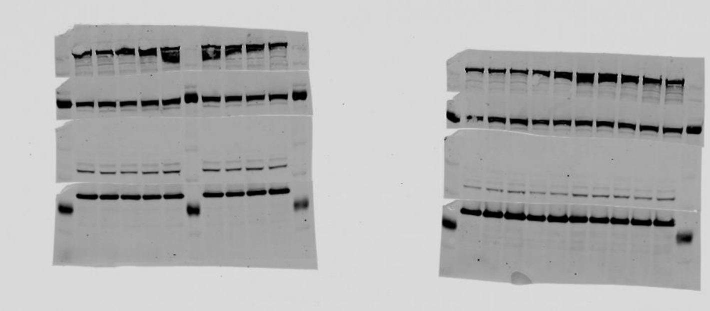

Figure 3D-ERK

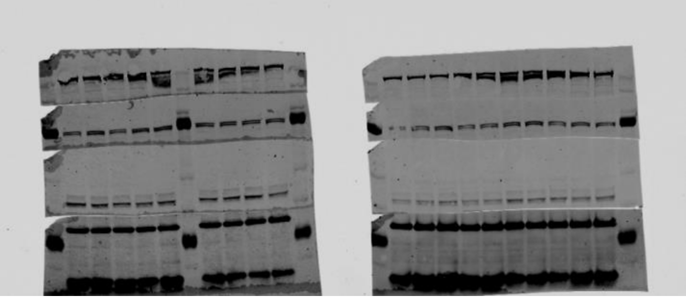

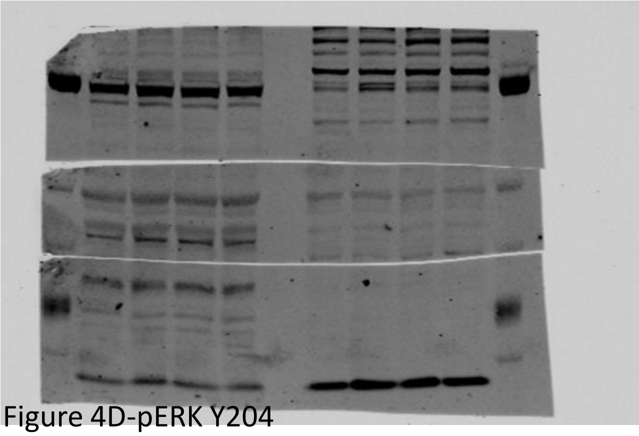
Figure 4D-pERK Y204

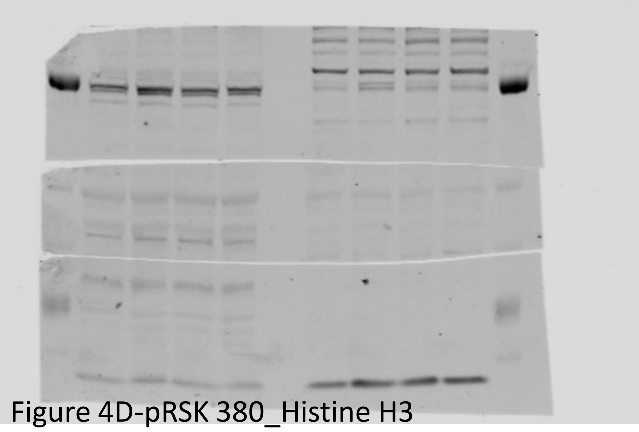
Figure 4D-pRSK 380_Histine H3

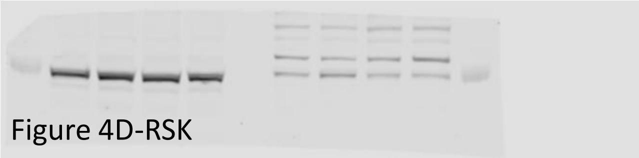
Figure 4D-RSK

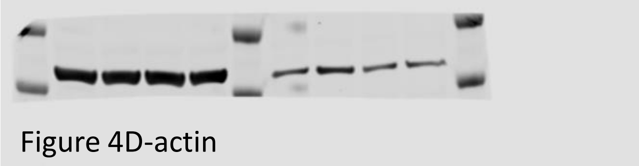
Figure 4D-actin

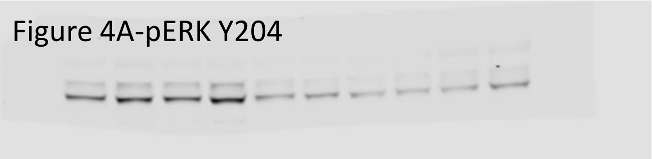
Figure 4A-pERK Y204

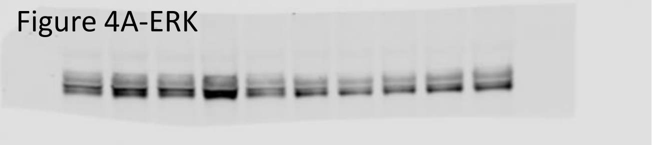
Figure 4A-ERK

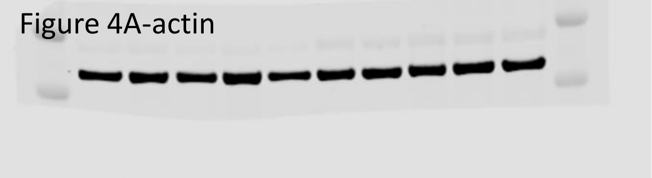
Figure 4A-actin

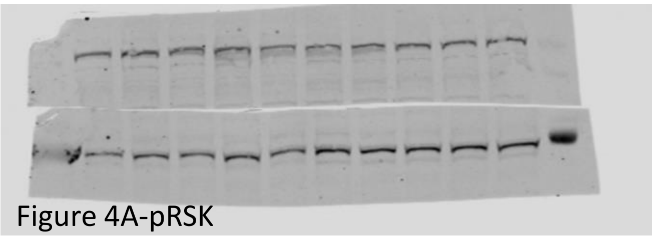
Figure 4A-pRSK

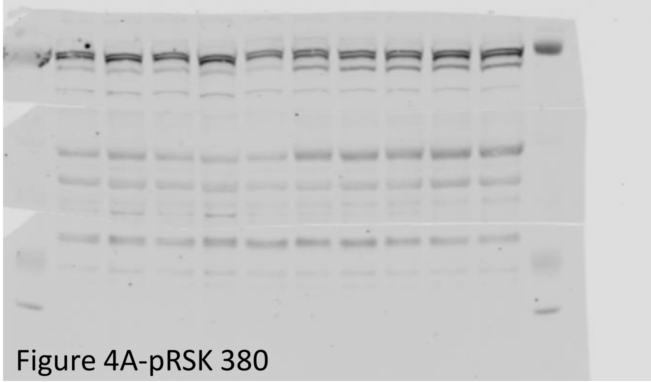
Figure 4A-pRSK 380

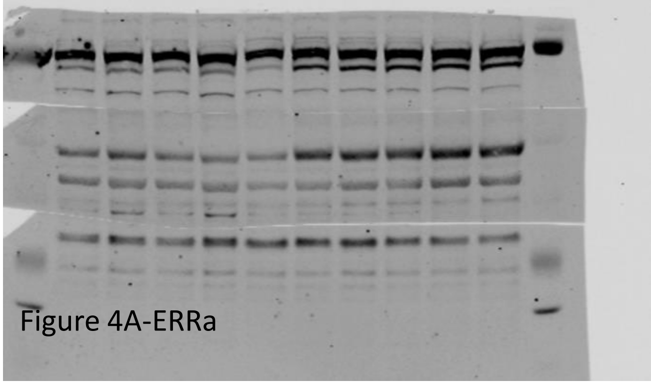
Figure 4A-ERRa

Figure 5D-PARPpERK Y204

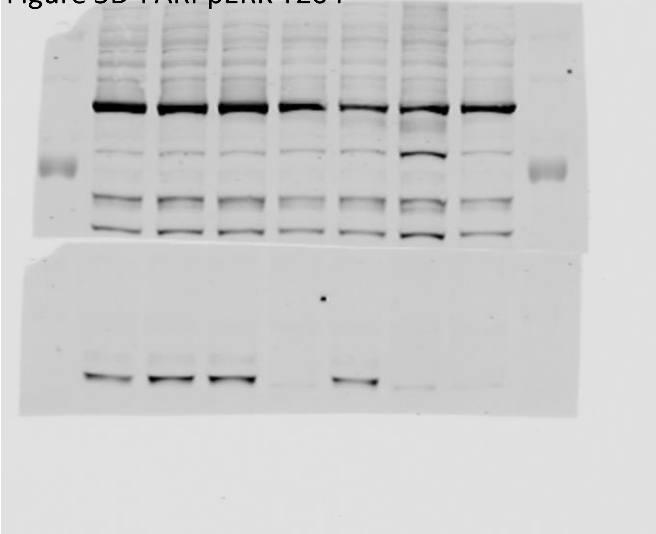

Figure 5D-ERRa

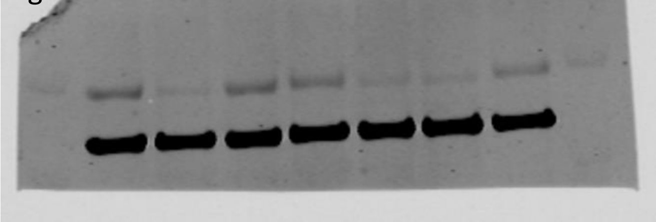

Figure 5D-actin

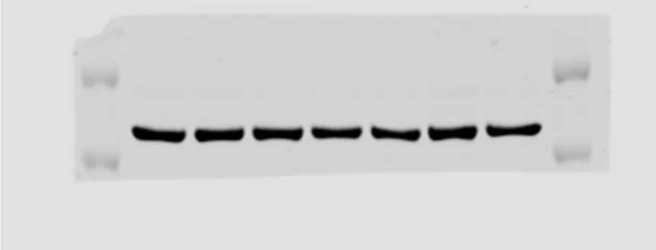

Figure 5D-pRSK 380

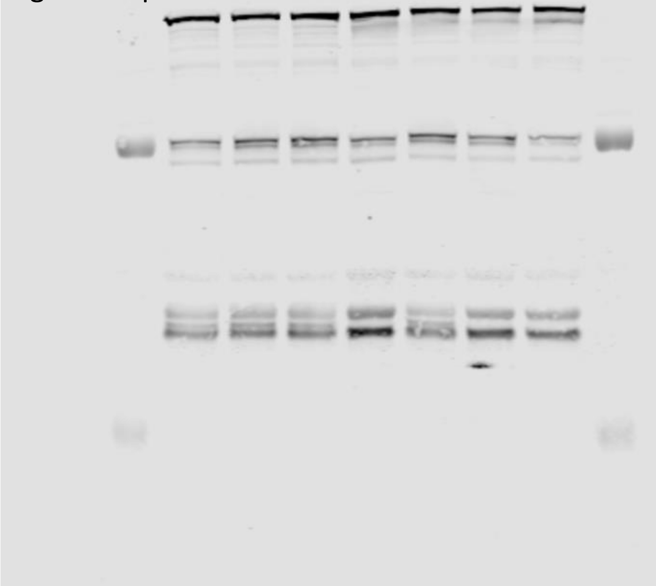

Figure 5C-pRSK 380

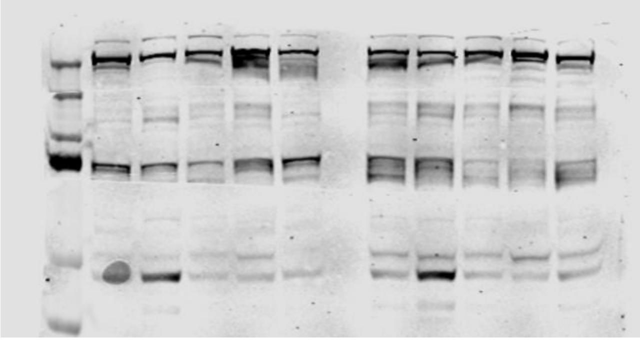

Figure 5C-pERK Y204

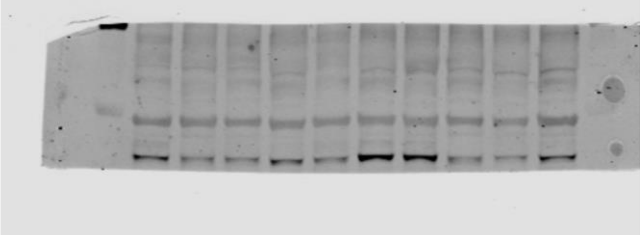

Figure 5C-ERRa

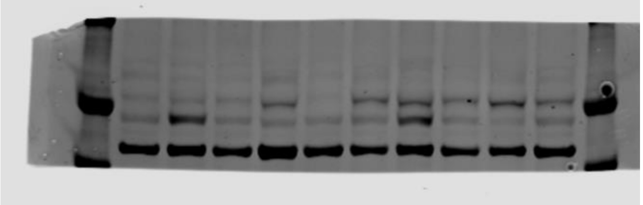

Figure 5C-actin

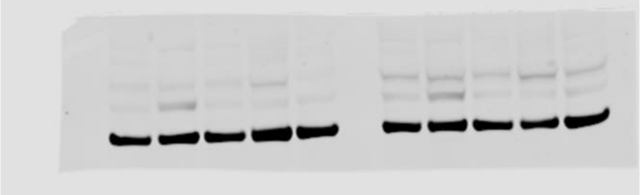

Supplement: S1 Raw images — (PDF) [file pone.0283047.s004.pdf]
